# How Do Spanish Hospitals Use Lean? Insights from a Multiple-Case Study

**DOI:** 10.3390/healthcare13233169

**Published:** 2025-12-04

**Authors:** Aneta Pawłowska-Hulbój, Bartosz Grucza, Michał Kozieł, Adam Kaniuk, Alicja Jakubowska, Wojciech Popiołek, Igor Pańkowski, Jaume Ribera, Jakub Batko, Mariusz Kowalewski, Wojciech Orzeł

**Affiliations:** 1 National Medical Institute of the Ministry of the Interior and Administration, 137 Wołoska Street, 02-507 Warsaw, Poland; 2 Department of Operations, Information and Technology Management, IESE Business School, Center for Research in Healthcare Innovation Management (CRHIM), University of Navarra, 08034 Barcelona, Spain

**Keywords:** lean healthcare management, emergency department, patients’ satisfaction, healthcare optimization

## Abstract

**Background/Objectives**: The European Society for Emergency Medicine reports that emergency department visits have increased by nearly 30% over the past decade, yet resources have not kept pace with this growing demand. Lean Healthcare Management has emerged as a promising approach to optimizing emergency department operations. This study aims analyze the specific Lean Healthcare Management interventions implemented across three major Barcelona hospitals. **Methods**: Three Barcelona hospitals were analyzed. Revision of the Lean Healthcare Management tools, hospital staff observation and focus groups with nurses, physicians, and administrators were performed to evaluate impact of Lean Healthcare Management interventions. A cumulative SWOT analysis was performed as a synthesis of individual responses and focus groups for the three included hospitals separately. **Results**: The average adherence scores to implemented Lean Healthcare Management solutions were 87% at Vall d’Hebron, 85% at Sant Joan de Déu, and 89% at Hospital Clínic de Barcelona. Implementation of Lean Healthcare Management led to 20% fewer cancelations of scheduled surgical procedures, decreased patient hospitalization times for targeted pathways (from 8 h to 70 min) and significant increase in patient satisfaction. All centers shared a common foundation in Value Stream Mapping. Implemented Lean Healthcare Management solutions were personalized for each hospital. **Conclusions**: Lean Healthcare Management’s effectiveness is contingent on aligning the Lean approach with the hospital’s specific mission, constraints, and patient population. This contextual dependency explains the variation in the tools adopted and the outcomes prioritized across the three analyzed hospitals.

## 1. Introduction

Emergency departments (EDs) are a vital component of healthcare systems, intended to provide immediate assistance to patients after emergencies or to those at risk of a quick health decline [1]. The European Society for Emergency Medicine reports that ED visits have increased by nearly 30% over the past decade, yet resources have not kept pace with this growing demand [2].

There are several causes of ED overcrowding, including increased input, longer throughput, delayed output, and other seasonal factors, associated with a significant increase in ED visits. One of the factors impacting ED increased input may be related to the concept of Health Debt generated by the COVID-19 pandemic, which led to a decreased number of planned hospitalizations, resulting in overloading the EDs with patients seeking medical attention [3]. In Italy, from 2019 to 2023, participation of non-urgent patients in all ED visits increased from almost 60% to over 70% [4]. One of the factors impacting the time of throughput is staff shortages and burnout. It was proven that the pandemic led to an increase in ED staff burnout in all three of its domains, with pooled prevalence increased from 35% before the pandemic to over 50% during the COVID-19 pandemic [5]. One of the main factors connected to delayed output is the hospital bed Access Block, which was especially seen during the pandemic [6,7]. In Spain, the COVID-19 pandemic gradually increased the number of emergency care requests (from 6 million in 2019 to 9 million in 2020). This increase was sustained in the most recent reports, which clearly present the significant overload of EDs in post-pandemic times. It should be noted that in pre-pandemic times, Catalonia had the third-highest number of ED admissions in the whole country, exceeding 194 emergencies/1000 inhabitants. However, despite the post-pandemic increase in the number of ED admissions across the entire country, ED admissions in Catalonia dropped by one-third [8,9]. It is unknown why such a significant shift was observed; however, it should be noted that during that period, implementation of lean healthcare management (LHM) was performed and finished in the main hospital centers in the capital of Catalonia, Barcelona.

LHM is a promising approach to optimizing ED operations [10]. It was proven that integration of lean management can improve emergency nurses’ workflow, reducing the number of unfinished nursing care from 73.4% to 39.6% with observed statistically significant patient satisfaction [11]. Additionally, it was proven to be superior in improving hospital waiting times in ED; as observed in a Vietnamese study, admission time to other hospital departments was reduced by half, time for pre-operative test results was reduced by 33% and patient satisfaction increased from 22.9 to 76.5% [12]. Additionally, a study performed in Poland revealed that the implementation of LHM led to time savings for nursing staff exceeding 368 h per month and 175 h per month for medical staff [13]. There are a lot of studies regarding LHM, analyzing its potential impact in simulations or discussing its optimization superiority; however, there is a lack of studies investigating its implementation impact on real hospital results and experience, especially in busy, publicly funded Mediterranean hospitals. Additionally, it is unknown how local organizational culture influences the mechanisms through which Lean affects outcomes.

This study aims to analyze the effect of the implementation of the specific LHM interventions across three major Barcelona hospitals.

## 2. Materials and Methods

### 2.1. Hospital Choice

The three hospitals—Vall d’Hebron University Hospital, Sant Joan de Déu Hospital, and Hospital Clínic de Barcelona—were chosen through purposive sampling. The selection criteria aimed to capture a range of LHM applications:

(1) Representation of Different Hospital Types—The sample includes a large public tertiary-care center, a specialized pediatric and obstetric hospital, and a leading academic research institution. This facilitates an examination of LHM adaptability across various patient populations, organizational sizes, and primary missions.
(2) Evidence of Ongoing LHM Implementation: All three hospitals had completed organization-wide LHM implementations, offering mature, stable systems for analysis rather than early-stage pilot programs.
(3) Presence of High-Volume, High-Pressure Settings: As major referral centers in a region with historically high ED utilization, these sites provide a rigorous context to evaluate LHM’s impact on operational efficiency under significant demand.
(4) Differences in Lean Methodologies: Each hospital used a unique set of LHM tools and philosophies, enabling a comparative analysis of different implementation strategies and their outcomes.
(5) Control for Macro-System Variables: By focusing on three hospitals within the same city and regional health system, the study accounted for overarching political, economic, and regulatory factors, allowing for a more explicit focus on organizational dynamics.

### 2.2. Study Methods

This study consisted of four main parts, aimed at collecting data needed to describe the LHM impact in the chosen hospitals altogether:

(1) Revision of the LHM tools implemented in each hospital based on workshops with the hospital management and the documentation.
(2) Hospital staff observation during work in situations in which the identified LHM tools were implemented.
(3) Focus groups with nurses, physicians, and administrators of the hospital separately.

Study visits were performed in November 2024. LHM tools implemented in each hospital were declared by the hospital management in charge of implementation, and then, based on the identified tools, administrative reports on hospital performance before and after the implementation were analyzed and compared to evaluate the impact of the intervention. Additionally, the protocols and standards of care created based on LHM were analyzed.

Hospital staff observation was performed during work in situations in which declared LHM tools were implemented to evaluate staff adherence to the declared LHM solutions and potential resistance points towards implemented solutions in everyday practice.

The population for hospital staff observation was chosen through purposive sampling. In each hospital, the following populations were observed:

(1) Physicians, working in a field in which the LHM intervention was implemented, who were employed before the LHM intervention’s implementation;
(2) Nurses, working in a field in which the LHM intervention was implemented, who were employed before the LHM intervention’s implementation;
(3) Physicians, working in a field in which the LHM intervention was implemented, who were employed after the LHM intervention’s implementation;
(4) Nurses, working in a field in which the LHM intervention was implemented, who were employed after the LHM intervention’s implementation.

During staff observation, adherence to declared LHM solutions was evaluated by two separate researchers on a scale from 1 to 10, where one marks no adherence and 10 marks complete adherence. Inter-Rater Reliability was assessed with Spearman’s Rho.

The population for focus groups was chosen through purposive sampling. The following inclusion criteria for focus group participants were implemented in this study:

(1) Participants who were employed before or after the LHM interventions, in equal numbers;
(2) Working as a physician, nurse, or administrator in a field in which LHM intervention was implemented;
(3) Willing to participate in focus groups.

In Vall d’Hebron University Hospital, 17 participants were selected, including four senior physicians (age range 45–60), three residents (age range 28–40), eight nurses (age range 25–55), and two hospital administrators (age range 35–60). Twenty staff members were invited to the study; three staff members refused to participate.

In Sant Joan de Déu Hospital, 15 participants were selected, including three senior physicians (age range 35–55), three residents (age range 27–34), seven nurses (age range 28–53), and two hospital administrators (age range 42–53). Twenty staff members were invited to the study; five staff members refused to participate.

In Hospital Clínic de Barcelona, 16 participants were selected, including three senior physicians (age range 35–52), three residents (age range 26–32), eight nurses (age range 32–49), and two hospital administrators (age range 49–57). Twenty staff members were invited to the study; four staff members refused to participate.

The focus group aimed at discussing the strong and weak points, opportunities, and threats of the implemented LHM solutions, and the impact of LHM implementation on everyday practice. The discussion was moderated by one of the researchers, who noted all suggestions on the whiteboard during the debate. The focus group was recorded. A detailed focus group guide for the moderator can be found in Table S1. After the discussion, each participant was asked to describe the strong and weak parts, opportunities, and threats of the implemented LHM individually. Responders’ identification data were anonymized for the analysis. A transcript was performed based on the focus group recording. Two separate researchers then analyzed each anonymized response and transcript by coding it to identify and interpret themes. The codebook can be found in the Table S2.

Inter-Rater Reliability was evaluated with test–retest reliability for each researcher and quantified with percent agreement. A cumulative SWOT analysis was performed as a synthesis of individual responses and focus groups for the three included hospitals separately.

## 3. Results

### 3.1. Overview of LHM Interventions and Adherence

A thematic analysis of the implemented Lean Healthcare Management (LHM) tools revealed distinct approaches across the three hospitals. However, all shared a common foundation in Value Stream Mapping (VSM) to identify and eliminate non-value-added steps. Staff adherence to the declared LHM solutions, as measured by independent observation, was generally high. The inter-rater reliability for adherence scores was strong (Spearman’s Rho = 0.89). The average adherence scores were 87% at Vall d’Hebron, 85% at Sant Joan de Déu, and 89% at Hospital Clínic de Barcelona.

### 3.2. Cross-Case Analysis of LHM Tools and Outcomes

The specific LHM interventions and their associated impacts on key performance metrics, with primary focus on changes directly affecting ED or, where specified, on hospital-wide processes that have a direct and documented effect on ED patient flow and output, are summarized in Table 1.

### 3.3. Thematic Analysis of LHM Interventions and Outcomes

The following section offers context for the data in Table 1, describing how the LHM tools were implemented and their effects as perceived by staff.

#### 3.3.1. Vall d’Hebron University Hospital

##### LHM Approach and Outcomes

The LHM implementation at Vall d’Hebron emphasized hospital-wide efficiencies that directly improved ED output. Horizontal process management formed interdisciplinary teams for specific diseases, which, according to focus group participants, resulted in “fewer laboratory examinations and shorter hospital stays” for these patient groups. This was recognized as a key factor in reducing access block for ED patients waiting for inpatient beds.

**Figure 1:**
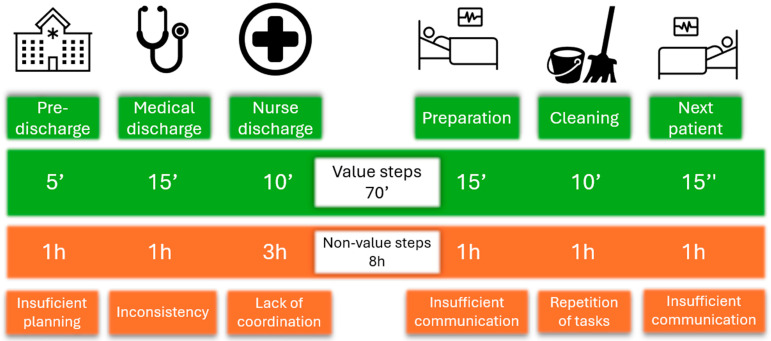
Value and non-value steps in preoperative and postoperative patient management.

Administrators specifically highlighted the introduction of buffer time slots in the operating room schedule as a way to accommodate emergency surgeries from the ED without disrupting the planned surgical schedule. Quantitative data from hospital reports confirmed a 20% decrease in planned procedure cancelations following this change. Optimization of preoperative patient management was achieved through a bottom-up strategy and brainstorming sessions, resulting in improvements that included the reduction in unnecessary administrative and logistical tasks, as well as the systemic elimination of inefficiencies (Figure 1).

Moreover, the implementation of a visual management system (red/yellow/green coding for surgical patients) and the digitalization of documentation helped reduce unnecessary staff movement and interruptions, allowing clinical staff to dedicate more time to patient care.

##### SWOT Analysis

A. Strengths
  ○ Enhanced Process Flow: Horizontal management and visual controls directly reduced delays and non-value-added steps, leading to fewer surgery cancelations and more efficient patient handling.
  ○ Resource Efficiency: Digital documentation and reorganized medical supplies optimized staff time and reduced physical movement.
  ○ Robust Emergency Integration: The buffer system for operating rooms effectively protected planned surgeries from emergency cases, directly improving ED-to-OR patient flow.
B. Weaknesses
  ○ Cultural Resistance: Focus groups indicated initial resistance from staff accustomed to traditional, department-centric hierarchies.
  ○ Implementation Overhead: The shift to horizontal management required significant initial coordination and restructuring.
  ○ Dependence on Coordination: The system’s efficiency relies heavily on seamless interdisciplinary collaboration, which can be a vulnerability.
C. Opportunities
  ○ Scalability of Low-Tech Tools: The success of visual management demonstrates the potential for wider application of low-cost, high-impact tools across other departments.
  ○ Data-Driven Refinement: The data collected from the new digital systems can be leveraged for further process optimization and predictive analytics.
  ○ Model for Large Centers: Its success as a large public tertiary-care center makes it a scalable model for similar hospitals.
D. Threats
  ○ Staff Burnout: The constant requirement for interdisciplinary coordination could contribute to fatigue if not managed carefully.
  ○ Budgetary Pressures: Future budget constraints could threaten the sustainability of the digital infrastructure and dedicated coordination roles.
  ○ Reliance on Key Personnel: The system’s stability may depend on the continued advocacy and effort of key change-makers.

#### 3.3.2. Sant Joan de Déu Hospital

##### LHM Approach and Outcomes

Sant Joan de Déu’s LHM approach, the E = MC^2^ model, is driven by structured projects that often start in the ED. Focus groups with nurses and physicians showed that process standardization, especially in blood sample collection and managing ED admissions, is seen as a way to cut down delays significantly. A key result was the very high Net Promoter Score (76.18), which the hospital management mostly credits to this ongoing, staff- and patient-led culture of improvement. Projects aimed at the ED, like developing specific protocols for patients with physical and mental disabilities, were frequently mentioned in focus groups as successful examples of boosting both efficiency and patient experience.

##### SWOT Analysis

A. Strengths
  ○ Exceptional Patient Satisfaction: The patient-centric model yielded a verifiable, high Net Promoter Score and positive patient feedback.
  ○ High Staff Engagement & Ownership: The bottom-up, project-based approach fostered strong buy-in and innovation among staff.
  ○ Structured Improvement Methodology: The E = MC^2^ model provides a clear, repeatable framework for continuous improvement.
B. Weaknesses
  ○ Model-Specific Complexity: The custom E = MC^2^ model may be more difficult to replicate or scale than generic Lean tools.
  ○ Potential for Project Silos: Continuous improvement through discrete projects could lead to a fragmented approach rather than a unified system-wide flow.
  ○ Resource Intensity: Maintaining a high level of staff engagement and running numerous projects requires significant ongoing organizational energy.
C. Opportunities
  ○ Brand Enhancement: The high patient satisfaction can be leveraged to strengthen the hospital’s reputation and attract more patients and funding.
  ○ Knowledge Export: The hospital can position itself as a center of excellence for patient-experience-focused Lean implementation.
  ○ Refinement of Specialized Care: The model is ideal for further optimizing care for complex and specialized patient populations.
D. Threats
  ○ Initiative Fatigue: Staff may become overwhelmed by the continuous cycle of projects and brainstorming sessions.
  ○ Dependence on Cultural Momentum: The success is deeply tied to a vibrant improvement culture, which can be fragile and susceptible to degradation with leadership or staff changes.
  ○ Measurement of Hard Outcomes: While patient satisfaction is high, the direct impact on some hard operational metrics (e.g., ED length of stay) is less explicitly documented than in other sites.

#### 3.3.3. Hospital Clínic de Barcelona

##### LHM Approach and Outcomes

The most integrated LHM system was observed at Hospital Clínic, where the ED used a strict Kanban-style patient flow system. Staff observations confirmed high adherence to this process, which organizes patient care into standardized 40-min cycles for both nurses and physicians. Focus group participants reported that this “first-in, first-out” approach reduced perceived chaos and variability during shifts. The real-time bed tracking system was identified by administrators as essential for managing ED output. By creating a “pool of available beds” and generating inventory summaries based on ED admissions, the hospital reported a significant reduction in the time ED patients spent waiting for an inpatient bed, thereby decreasing overall ED length of stay.

Several enhancements of performance indicators and support services have been implemented:

- Improved pharmacy management and medication distribution (e.g., using systems for cytostatic or anti-infective therapy).
- Implementation of analytical applications, including SAP automation, to avoid duplicate procedures and conduct cost reviews of medical procedures.
- Optimization of demand for imaging studies and their interpretation.
- Redirecting hospitalized patients to outpatient care and maintaining continuous collaboration with emergency services.
- Ongoing price negotiations with suppliers of materials and medications (e.g., reducing prosthesis costs by 30% through group purchasing), as well as IT services and medical equipment.
- Optimization of consumables’ usage through implementing consumption monitoring and the allocation of specific supply batches to departments, reducing waste.

##### SWOT Analysis

A. Strengths
  ○ Maximized Operational Efficiency: The Kanban system and real-time bed tracking created a highly predictable and efficient patient flow, directly reducing variability and delays.
  ○ Strong Technological Integration: IT systems are deeply embedded in the Lean processes, providing data for real-time management and decision-making.
  ○ Significant Cost Control: A systematic approach to supply chain and process optimization yielded verifiable financial benefits.
B. Weaknesses
  ○ High Technological Dependency: The system’s performance is heavily reliant on a complex, functioning IT infrastructure.
  ○ Rigidity: The highly standardized processes (e.g., 40-min cycles) may lack the flexibility to handle extreme or unpredictable patient surges effectively.
  ○ Potential for Staff Alienation: The “factory-like” precision of the ED system could be perceived as dehumanizing by some staff or patients if not balanced with a caring culture.
C. Opportunities
  ○ Leadership in Healthcare IT: The hospital is well-positioned to pioneer the integration of AI and advanced analytics for predictive patient flow management.
  ○ Benchmark for Throughput: It can serve as a benchmark for other institutions seeking to maximize efficiency and reduce costs in high-volume settings.
  ○ Data-Driven Procurement: The success in supply chain optimization can be expanded to other areas of hospital spending.
D. Threats
  ○ IT System Failure: Any significant downtime in the bed-tracking or management systems would severely disrupt operations.
  ○ High Initial Investment: The technological backbone required for this model presents a significant barrier to adoption for less-resourced hospitals.
  ○ Workforce Skill Gaps: Requires staff who are both clinically proficient and comfortable working within a highly structured, technology-driven environment.

## 4. Discussion

### 4.1. Summary of Findings

This multiple-case study provides a exploration of LHM implementation in the three leading Barcelona hospitals. Our findings illustrate that while LHM is a highly adaptable strategy, its application, benefits, and challenges are profoundly shaped by the local organizational context.

A primary finding of our study is that there is no universal blueprint for LHM; its effectiveness is contingent on aligning the Lean approach with the hospital’s specific mission, constraints, and patient population. This contextual dependency explains the variation in the tools adopted and the outcomes prioritized across the three sites.

Vall d’Hebron, a large tertiary center, focuses on hospital-wide flow by implementing horizontal process management and OR buffer slots to address the critical issue of access being blocked from the ED. Their success in reducing surgery cancelations by 20% aligns with studies showing that downstream bottlenecks often limit ED performance and that improving flow across departmental silos is essential [6,7].

Sant Joan de Déu, a specialized pediatric hospital, adopted a patient-focused, project-driven model (E = MC^2^). Their outstanding Net Promoter Score (76.18) highlights that in some settings, LHM’s most significant influence can be on experience and satisfaction, benefits often seen more quickly than traditional throughput measures in complex care environments.

Hospital Clínic implemented the most integrated and technologically advanced system, with its Kanban patient flow and real-time bed tracking, creating a highly controlled, efficient environment. The significant reduction in hospital stays and supply costs highlights the potential of a systemic, technology-driven approach, but also its vulnerability to IT failures and rigidity.

This heterogeneity challenges the notion of “best practices” in LHM and instead argues for “best-fit” strategies. A tool that succeeds in one context may fail in another if the underlying organizational readiness and problem profile do not align.

Below, the SWOT analysis synthesizes the key findings from Vall d’Hebron University Hospital, Sant Joan de Déu Hospital, and Hospital Clínic de Barcelona to provide a holistic view of LHM implementation impact in the context of EDs.

A. Strengths
  ○ Enhanced Patient Flow and Throughput: The implementation of standardized workflows directly reduces ED variability, minimizes patient batching, and creates a more predictable, first-in-first-out process. This leads to reduced waiting times and decreased ED length of stay.
  ○ System-Wide Efficiency Gains: LHM tools like Value Stream Mapping and horizontal process management successfully identify and eliminate non-value-added steps not just within the ED, but also at critical interfaces.
  ○ Improved Resource Utilization: Initiatives such as digital documentation, pre-packed medical supplies, and optimized supply chains reduce waste and free up staff time, allowing ED personnel to focus more on direct patient care.
  ○ High Staff Engagement and Patient Satisfaction: The bottom-up, project-based approach fosters a sense of ownership among staff. At the same time, the focus on patient-centric care directly translates into measurable patient satisfaction outcomes.
B. Weaknesses
  ○ Significant Implementation Overhead: Successful LHM requires a profound cultural shift away from traditional hierarchies and siloed departments. Gaining full staff buy-in is time-consuming and can face considerable resistance, making the initial implementation phase complex and challenging.
  ○ Dependence on Enabling Factors: The most advanced efficiency gains are often reliant on technology (e.g., IT systems for bed tracking) and continuous coordination. In resource-constrained settings, this dependence can be a critical vulnerability.
  ○ Sustainability Challenges: Maintaining the gains requires ongoing monitoring, training, and staff engagement. There is a persistent risk of reverting to old habits, especially without dedicated process improvement teams or in the face of staff turnover.
  ○ Potential for Rigidity and Alienation: Highly standardized processes (e.g., strict time cycles) may struggle to adapt to extreme, unpredictable patient surges. Furthermore, an excessive focus on efficiency can be perceived by staff as dehumanizing if not balanced with a strong culture of care.
C. Opportunities
  ○ Scalability of Low-Tech Solutions: The demonstrated success of visual management and physical Kanban boards shows that significant ED improvements can be achieved without massive IT investments, making LHM adaptable to a wide range of hospital budgets.
  ○ Technological Integration: The foundation laid by LHM creates a perfect platform for integrating advanced digital tools (e.g., AI-driven predictive analytics for patient flow, SAP automation) to enhance decision-making and efficiency further.
  ○ Strengthening Care Pathways: LHM provides a framework to build robust collaborative models with primary care and other community services. This can help redirect non-urgent cases away from the ED, directly addressing a root cause of overcrowding.
  ○ Policy and Competitive Advantage: Growing emphasis on healthcare efficiency can drive funding and policy support for LHM initiatives. Hospitals can also leverage improved performance and patient satisfaction as a key differentiator.
D. Threats
  ○ Resource Constraints: Tight budgets and staffing shortages—common in healthcare systems globally—pose the greatest threat. LHM relies on engaged personnel and some initial investment; without these, implementation can fail or deliver suboptimal results.
  ○ Cultural and Hierarchical Resistance: Clinicians may perceive LHM as a top-down, cost-cutting measure rather than a quality improvement tool. Deep-seated hierarchical structures can stifle the bottom-up engagement essential for long-term success.
  ○ External System Pressures: Seasonal demand surges (e.g., flu season) and pandemics can overwhelm even the most optimized, standardized ED processes, highlighting the need for built-in flexibility.
  ○ Systemic Inertia: In publicly funded or highly bureaucratic systems, slow decision-making and regulatory hurdles can delay the adoption of new processes and technologies, causing LHM initiatives to lose momentum.

### 4.2. Comparison with Previous Reports

Our findings both corroborate and complicate the existing evidence on LHM. The reported benefits—such as reduced waiting times, improved process standardization, and high staff adherence—are consistent with numerous studies on Lean in healthcare [11,12,13]. For instance, the time savings for nursing and medical staff we observed are in line with quantitative results from a Polish study, while the focus on patient satisfaction echoes positive outcomes from Vietnam [12,13].

However, our cross-case analysis reveals nuances often absent from single-site success stories. The cultural resistance and significant implementation overhead we identified at Vall d’Hebron are well-documented but frequently underemphasized barriers [14]. The potential for initiative fatigue at Sant Joan de Déu serves as a critical reminder that continuous improvement requires continuous energy, which is a finite resource in often-overstretched healthcare systems. Furthermore, the rigidity and technological dependency at Hospital Clínic pose essential questions about the scalability of such models to less-resourced settings or their resilience during unpredictable demand surges, a common critique of highly standardized systems in dynamic environments [15].

### 4.3. Implications for Policy and Practice

The successful implementation of Lean Healthcare Management (LHM) in Barcelona’s hospitals provides valuable lessons for other EDs, especially in regions with ED difficulties, including Central and Eastern Europe (CEE). However, significant adaptations are needed to account for regional differences in resources, infrastructure, and healthcare systems [15,16,17,18]. While the core principles of LHM—eliminating waste, optimizing workflows, and enhancing patient value—remain universally applicable, the specific strategies must be carefully tailored to address the unique challenges faced by CEE countries, including tighter budgets, workforce shortages, aging infrastructure, and more hierarchical organizational cultures [15,16,17,18].

A summary of the proposed LHM adaptation in various challenges of CEE EDs is presented in Table 2.

The technological divide between Western and Eastern Europe presents both challenges and opportunities [17,19,20]. Low-tech solutions, including the Kanban system or visual management system, may be constructive for CEE hospitals that often face budget constraints that limit major technological upgrades [21].

In CEE EDs severe staffing shortages and higher burnout rates are observed, which may impact potential LHM implementation associated with increased workload [21,22]. Organizational culture might be one of the main barriers to LHM adoption in CEE countries. The region’s healthcare systems tend to be more hierarchical and resistant to change compared to their Western European counterparts [14]. To overcome this, the use of quick-win strategies, associated with tangible benefits to staff, such as reduced paperwork or more predictable work schedules, might positively impact sustainability and staff support in implementing changes.

### 4.4. Future Research and Limitations

This study has several limitations that should be acknowledged. First, selecting three high-performing, innovative hospitals in a single urban area limits how broadly the findings can be applied, especially to smaller, rural, or less-resourced settings. Second, relying on management-declared tools, internal performance metrics, and staff self-reports introduces the risk of social desirability and recall bias. We did not independently gather or verify patient-level outcome data, so our claims about clinical effectiveness are suggestive rather than conclusive. Third, the cross-sectional design offers a snapshot of post-implementation success but does not address the long-term sustainability of these LHM initiatives. Future research should adopt longitudinal, mixed-methods approaches to monitor how LHM implementations evolve. Studies should specifically examine the cost-effectiveness of different LHM tools in low-resource settings and aim to connect process changes with strong, patient-centered clinical outcomes. Additionally, research is needed to explore why some LHM implementations fail or do not sustain, providing a more balanced and practical evidence base.

## 5. Conclusions

This study shows that LHM is a flexible strategy that is capable of transforming ED operations, even in resource-limited settings. The experiences of hospitals in Barcelona highlight the effectiveness of interventions such as Value Stream Mapping, interdisciplinary teams, and technological integration in reducing waste, improving workflows, and increasing patient satisfaction. For CEE EDs, successful implementation requires focusing on low-cost tools, engaging staff, and using incremental steps to overcome financial and cultural challenges.

## Figures and Tables

**Table 1:** Cross-Case Analysis of Lean Tools and Documented Outcomes in Barcelona Hospital Emergency Departments.

Hospital	Primary LHM Tools Implemented	Key Documented Outcomes (Pre- vs. Post-LHM)
Vall d’Hebron University Hospital	Buffer Time Slots (in OR schedules) Visual Management (Color-coded patient tracking) Horizontal Process Management (Interdisciplinary teams for specific patient pathways)	20% fewer cancelations of scheduled surgical procedures. Decreased patient hospitalization times for targeted pathways (from 8 h to 70 min). Reduction in unnecessary staff movement and transportation of documentation.
Sant Joan de Déu Hospital	E = MC^2^ Model (Structured, project-based improvement) Process Standardization (e.g., blood sample collection, ED admission) Patient-Centric Redesign (e.g., protocols for patients with disabilities)	Net Promoter Score of 76.18 from patients and families (2024). Reduction in waiting times for blood sample collection and specialty appointments. High staff engagement was reported in focus groups.
Hospital Clínic de Barcelona	Kanban System for Patient Flow (Structured 40-min cycles for doctor/nurse work) 6S Workplace Organization Real-Time Bed Tracking (“Pool of available beds”) Buffer Scheduling for ED/OR interface	Reduced variability and patient batching in the ED. Significant decrease in hospital stays. 30% reduction in prosthesis costs through optimized supply chain.

ED—emergency department; OR—operating room; LHM—lean healthcare management.

**Table 2:** A summary of the proposed LHM adaptation in various challenges of Central and Eastern European Emergency Departments.

Challenge in CEE EDs	Lean Adaptation Strategy	Implementation Approach	Expected Benefits
Budget constraints	Low-tech visual management	Color-coded patient tracking boards, physical Kanban systems	Improved workflow transparency without costly IT investments
Workforce shortages	Cross-training & staff engagement	Bottom-up improvement teams, multi-skilling programs	Increased operational flexibility, better resource utilization
Hierarchical culture	Pilot projects with leadership support	Start small in one unit, demonstrate quick wins to build buy-in	Gradual cultural shift, reduced resistance to change
Aging infrastructure	Process standardization	Value Stream Mapping for high-volume areas (triage, diagnostics)	Reduced variability, eliminated non-value-added steps
Seasonal demand surges	Flexible surge protocols	Adjustable staffing models, modular care spaces	Better capacity management during peak periods
Limited IT resources	Basic digital solutions	Leverage existing medical records for simple tracking, partner with local tech providers.	Affordable workflow improvements
Patient flow bottlenecks	Horizontal process management	Integrated care pathways, improved ED–primary care coordination	Reduced admission/discharge delays
Sustainability concerns	Dedicated process teams	Establish small improvement units, and regular training programs	Maintained momentum despite staff turnover
Financial constraints	Phased high-region of interest implementation	Prioritize interventions with the fastest returns (e.g., bed turnover)	Creates a self-funding improvement cycle
Overcrowding	Buffer concepts	Adapted surgical scheduling approaches for ED surge capacity	Reduced cancelations, better emergency case management

## Data Availability

Data is available from the corresponding author upon reasonable request.

## Supplementary Materials

The following supporting information can be downloaded at https://www.mdpi.com/article/10.3390/healthcare13233169/s1: Table S1. Focus group guide; Table S2. Codebook.

## Abbreviations

CEE	Central and Eastern Europe
ED	Emergency Department
LHM	Lean Healthcare Management
OR	Operating Room
SWOT	Strengths, Weaknesses, Opportunities, Threats
VSM	Value Stream Mapping
