# Supplementary material for: How Do Spanish Hospitals Use Lean? Insights from a Multiple-Case Study
Source: Healthcare (Basel). 2025 Dec 4;13(23):3169. doi: 10.3390/healthcare13233169 (PMC12692682; doi:10.3390/healthcare13233169)
Supplement: Supplementary file 1 [file healthcare-13-03169-s001.zip › healthcare-3875900-supplementary.pdf]

Table S1. Focus group guide

Focus Group Guide: Experiences with Lean Healthcare Management (LHM) Implementation

Moderator: [Researcher's Name]

Note-taker/Scribe: [Assistant's Name]

Date: [Date of Focus Group]

Location: [Hospital Name, Private Room]

Overall Purpose: To explore the experiences, perceptions, and insights of healthcare staff (physicians, nurses, administrators) regarding the implementation and impact of Lean Healthcare Management (LHM) tools and principles in their Emergency Department and associated hospital units.

I. Introduction (Approx. 10 minutes)

Moderator Script:

"Good morning/afternoon, and thank you all so very much for taking the time to participate in this discussion today. My name is [Researcher's Name], and I will be moderating our session. With me is [Assistant's Name], who will be taking notes.

1. Welcome & Purpose:

"We are researchers conducting a study on the implementation of Lean Healthcare Management, or LHM, here at [Hospital Name]. The goal of this focus group is to learn from you, the experts on the front lines, about your experiences with these changes. We are interested in hearing about what has worked well, what has been challenging, and how these changes have affected your daily work and patient care."

2. Ground Rules:

"Before we begin, I'd like to set a few ground rules to make our conversation productive:

1. No Right or Wrong Answers: We are here to hear your honest opinions and experiences. There are no right or wrong answers. We are interested in all perspectives, both positive and negative.
2. One Person Speaks at a Time: Please speak one at a time so our note-taker can accurately capture everything.
3. Confidentiality: What is said in this room stays here. We ask that you all respect each other's privacy and do not repeat what is shared outside of this group.
4. Recording: With your permission, we would like to audio-record this session to ensure we don't miss any of your valuable comments. The recordings will be used only for research analysis and will be stored securely. All identifying information will be removed from the final transcripts.
5. Participation: We would like everyone to participate. It's perfectly okay to disagree; different viewpoints are exactly what we're looking for. I may call on some of you if I feel we haven't heard from everyone, but please feel free to jump in at any time."

3. Consent & Recording:

"Does anyone have any questions about these rules or the study in general? [Pause for questions]. If you are comfortable with being recorded, please verbally confirm 'yes' now so we have it on tape."

(Start audio recording)

"Thank you. Let's begin by going around the room. Please state your professional role (e.g., nurse, physician, administrator) and how long you have been working at this hospital."

II. Opening & Broad Impressions (Approx. 15 minutes)

Objective: To get participants comfortable speaking and to gather initial, top-of-mind impressions of the LHM changes.

Moderator Script/Questions:

1. "To start, thinking about the Lean management changes that have been introduced here—such as [Mention 1-2 specific tools, e.g., the new patient flow system, visual management boards, interdisciplinary teams]—what is the first word or short phrase that comes to your mind?"

2. "Can you briefly describe, in your own words, what you understand the main goals of these LHM changes to be?"

3. "Looking back, what was the general feeling among staff when these new processes were first introduced?"

III. Core Discussion: Deep Dive into the LHM Experience (Approx. 45 minutes)

Objective: To explore the specific strengths, weaknesses, and impacts of the LHM implementation in detail.

Moderator Script/Questions:

A. Strengths & Positive Outcomes:
   1. "Let’s talk about what has worked well. Can you describe a specific situation where you felt one of the new LHM tools or processes made a positive difference?"
   2. Probe for: Impact on patient care, your own workflow, teamwork, or communication.
   3. "From your perspective, what is the single biggest strength or benefit that has come from this Lean approach?"

B. Challenges & Weaknesses:
   1. "Now, let's talk about the other side. What has been the most significant challenge or difficulty in using these new systems?"
   2. Probe for: Specific tools that are frustrating, increased workload at certain points, resistance from colleagues, or technical issues.
   3. "Were there any aspects of the implementation that you feel could have been handled better? For example, the training, communication, or support during the transition?"

C. Impact on Daily Practice & Patient Care:
   1. "How have these changes specifically altered your daily routines and responsibilities?"
   2. "From your vantage point, what has been the impact on patients and their families? Have you noticed any changes in their experience, satisfaction, or the care they receive?"

3. Probe for both positive and negative observations.

D. Sustainability & The Future:
1. "Do you believe these changes are here to stay? Why or why not?"
2. "What would be needed to ensure these improvements are sustained over the long term?"
3. "What opportunities do you see for further improving efficiency or patient care using this Lean mindset in the future?"

IV. Conclusion and Final Reflections (Approx. 10 minutes)

Objective: To ensure all voices are heard and to capture any final, crucial insights.

Moderator Script/Questions:

1. "We are nearing the end of our time. Is there anything important that we haven't discussed that you feel we should know about your experience with these changes?"
2. "Finally, if you were to give one piece of advice to another hospital about to start a similar Lean journey, what would it be?"

V. Closing & Next Steps

Moderator Script:

"This concludes our formal discussion. Thank you again for your candor and for sharing your incredibly valuable insights. Your contributions are essential to this research.

We will now stop the audio recording. Thank you once more for your time and participation."

(Stop audio recording)

As a final step, we would like you to individually and anonymously write down what you believe are the strong points, weak points, opportunities, and threats of the implemented LHM system. We will provide you with a sheet of paper for this.

[Distribute paper and pens. Allow 10 minutes for writing].

Table S2. Codebook.

Theme	Sub-theme	Statement focus
Perceived Benefits and Strengths - Statements that describe the positive outcomes, advantages, and successful aspects of LHM implementation from the perspective of staff and the organization.	**Process Efficiency**	Improvements in workflow, reduction of delays, and elimination of unnecessary steps.
	**Resource Optimization**	Better utilization of staff time, physical space, and medical supplies.
	**Patient-Centered Care**	Enhancements in the quality of care, patient experience, and holistic treatment.

	**Staff Empowerment & Engagement**	Feelings of ownership, involvement in decision-making, and increased job satisfaction among staff.
	**Cost and Time Reduction**	Tangible reductions in operational costs, wasted time, and resource consumption.
**Implementation Challenges and Weaknesses - Statements that describe the difficulties, obstacles, and negative aspects encountered during or after the implementation of LHM.**	**Implementation Complexity**	The difficulty of initiating change, requiring significant effort, training, and cultural shift.
	**Cultural and Hierarchical Resistance**	Pushback from staff due to comfort with existing routines, professional silos, or top-down resistance.
	**Technological Dependencies & Gaps**	Challenges related to reliance on IT systems or the inadequacy of existing technological infrastructure.
	**Sustainability Concerns**	Worries about maintaining the new practices long-term and the risk of reverting to old methods.
	**Increased Initial Workload**	The perception that setting up and adapting to LHM initially creates more work for staff.
**Enablers and Opportunities for Success - Statements that identify factors, strategies, or future possibilities that support or could enhance the successful adoption and scaling of LHM.**	**Leadership and Management Support**	The critical role of committed leadership in championing and resourcing the LHM initiative.
	**Staff Involvement & Bottom-Up Approach**	The value of engaging frontline staff in the design and problem-solving process.
	**Effective Training and Communication**	The importance of clear, ongoing training and transparent communication throughout the process.

	**Scalability and Adaptability**	The perception that LHM principles or specific tools can be applied to other areas or settings.
	**Technological Integration**	The opportunity to use existing or new digital tools to support further and automate Lean processes.
**External Threats and Contextual Barriers**	**Resource Constraints**	Limitations imposed by budget cuts, staffing shortages, or lack of physical space.
	**Variable and Unpredictable Demand**	Challenges posed by seasonal surges, pandemics, or other fluctuations in patient volume.
	**Regulatory and Bureaucratic Hurdles**	Slow decision-making or implementation barriers caused by healthcare system regulations or administrative red tape.
	**Staff Burnout and Turnover**	The risk that pre-existing high levels of staff fatigue and turnover will undermine LHM efforts.
